# Discrepancies in Serology-Based and Nucleic Acid-Based Detection and Quantitation of Tomato Spotted Wilt Orthotospovirus in Leaf and Root Tissues from Symptomatic and Asymptomatic Peanut Plants

**DOI:** 10.3390/pathogens10111476

**Published:** 2021-11-12

**Authors:** Pin-Chu Lai, Mark R. Abney, Yi-Ju Chen, Sudeep Bag, Rajagopalbabu Srinivasan

**Affiliations:** 1 Department of Entomology, University of Georgia, Griffin, GA 30223, USA; pclai@uga.edu (P.-C.L.); yijuchen@uga.edu (Y.-J.C.); 2 Department of Entomology, University of Georgia, Tifton, GA 31793, USA; mrabney@uga.edu; 3 Department of Plant Pathology, University of Georgia, Tifton, GA 31793, USA; sudeepbag@uga.edu

**Keywords:** *Arachis hypogaea* L., spotted wilt, serological detection, overestimation, tissue type, virus accumulation

## Abstract

Thrips-transmitted tomato spotted wilt orthotospovirus (TSWV) causes spotted wilt disease in peanuts. A serological test (DAS-ELISA) is often used to detect TSWV in peanut leaf samples. However, in a few studies, DAS-ELISA detected more TSWV infection in root than leaf samples. It was not clear if the increased detection was due to increased TSWV accumulation in root tissue or merely an overestimation. Additionally, it was unclear if TSWV detection in asymptomatic plants would be affected by the detection technique. TSWV infection in leaf and root tissue from symptomatic and asymptomatic plants was compared via DAS-ELISA, RT-PCR, and RT-qPCR. TSWV incidence did not vary by DAS-ELISA, RT-PCR, and RT-qPCR in leaf and root samples of symptomatic plants or in leaf samples of asymptomatic plants. In contrast, significantly more TSWV infection and virus load were detected in root samples of asymptomatic plants via DAS-ELISA than other techniques suggesting that DAS-ELISA overestimated TSWV incidence and load. TSWV loads from symptomatic plants via RT-qPCR were higher in leaf than root samples, while TSWV loads in leaf and root samples from asymptomatic plants were not different but were lower than those in symptomatic plants. These findings suggested that peanut tissue type and detection technique could affect accurate TSWV detection and/or quantitation.

## 1. Introduction

Spotted wilt disease of peanut (*Arachis hypogaea* L.) is caused by the tomato spotted wilt orthotospovirus (TSWV), and TSWV is exclusively transmitted by nine thrips species in the order Thysanoptera and family Thripidae [1,2,3]. Tomato spotted wilt orthotospovirus is the type species in the genus *Orthotospovirus*, family *Tospoviridae*, and order *Bunyavirales* [3]. Besides peanut, the host range of TSWV includes over a thousand plants species in 15 monocot families and 69 dicot families [4]. TSWV is the most impactful virus affecting peanuts in the southeastern U.S. [1].

TSWV is an important yield-limiting factor and is ubiquitous across major peanut-producing states in the Southeast since the first report of spotted wilt in peanut in 1971 in the U.S. [5,6,7,8,9,10]. In Georgia, the top U.S. peanut-producing state, the annual peanut yield loss to TSWV was estimated at over USD 20 million from 2015 to 2018 [11,12,13,14,15].

The most prominent symptoms of spotted wilt disease are concentric ring spots and chlorosis on peanut foliage. In severe cases of TSWV infection, peanut plants are stunted or even dead. Other symptoms such as small or misshaped pegs, pods, kernels, and reddish discoloration of the seed coats can also be found on below-ground plant parts [1,9,16,17]. Visual assessment of symptoms is commonly used to evaluate TSWV incidence and severity [18,19,20]. However, biotic or abiotic factors, including temperature/water stress, nutrient deficiency, pest infestation, and other pathogen infections, can make symptoms induced by TSWV infection difficult to recognize. Different detection techniques are used to confirm the presence of TSWV. Among the available techniques, serology-based double antibody sandwich enzyme-linked immunosorbent assay (DAS-ELISA) is most commonly used to detect TSWV in peanut tissues in the laboratory, while ImmunoStrip^®^ (Agdia, Elkhart, IN, USA) test is largely used for on-farm testing. Another less frequently used laboratory technique is nucleic acid-based reverse-transcription polymerase chain reaction (RT-PCR) [21,22,23,24,25,26,27,28]. Reverse-transcription quantitative polymerase chain reaction (RT-qPCR) is an additional technique that is available to detect TSWV and/or to quantitate TSWV loads in peanut tissue samples [29,30,31].

TSWV incidence is often evaluated to assess TSWV susceptibility of cultivars/genotypes in breeding trials and the efficacy of TSWV management practices, and such evaluations are facilitated by laboratory detection methods [25,28,32,33,34]. DAS-ELISA utilizes TSWV-specific monoclonal/polyclonal antibodies to capture TSWV, which is then conjugated with an enzyme that induces a colorimetric response in the presence of a substrate [35,36]. DAS-ELISA has become the standard plant virus detection method due to its relatively low cost and scalability for larger sample sizes [37,38,39]. RT-PCR involves reverse-transcription to synthesize complementary DNA (cDNA) from total RNA and uses TSWV-specific primers to amplify the targeted TSWV gene from cDNA [23,40]. RT-PCR is more specific and sensitive than DAS-ELISA but is less frequently used, as it requires total RNA extraction from peanut tissue samples, specialized equipment, advanced operational skills, and is more expensive than DAS-ELISA [37,41]. RT-qPCR differs from RT-PCR in that the PCR product is detected in real-time during amplification. This is commonly achieved using non-specific fluorescent dyes that intercalate with double-stranded DNA or complementary fluorescent probes that bind to the amplicon by base-pairing [42]. The amount of the amplicon (i.e., TSWV capsid protein gene) can be determined by absolute/relative quantitation [43,44].

TSWV infection in peanuts is systemic, and TSWV has been detected in multiple peanut plant tissues, including leaves, pegs, pods, and roots [26,27]. Peanut leaf tissue is typically used for TSWV detection via DAS-ELISA; however, one study indicated that the distribution of TSWV in peanut leaves was not uniform [24]. In a few other studies, TSWV was detected at a higher percentage in root than leaf tissue samples [16,22,25,27]. This led to the presumption that root tissue could serve as a better reservoir for TSWV than leaf tissue. Nevertheless, in planta movement and accumulation of TSWV in peanuts is not completely understood. It is not clear if the difference in TSWV detection between root and leaf tissue samples via DAS-ELISA is due to higher virus loads in root versus leaf tissue or if the difference is the result of the DAS-ELISA producing false positives when root tissue samples are tested. Alternatively, it is also not clear if RT-PCR testing is resulting in the detection of false negatives with root tissue samples due to the presence of PCR inhibitors. A more recent study compared TSWV detection by DAS-ELISA and RT-PCR using root samples but not leaf samples from randomly collected field-grown peanut plants and found good congruence between the two methods [22]. However, no study has compared DAS-ELISA detection of TSWV in symptomatic leaf and root tissue samples with RT-PCR detection. TSWV infection is also detected in asymptomatic plants, especially often in diploid peanut species [21,25,45,46]. DAS-ELISA-based virus detection in leaf and root tissue samples could be affected by numerous extraneous factors and warrants comparison with other detection methods to assess whether DAS-ELISA-based TSWV testing in asymptomatic plant tissue samples produces accurate results or overestimates due to the detection of false positives [47,48,49,50]. Thus far, no such comparison has been performed with asymptomatic peanut tissue samples.

This study compared TSWV detection efficiency and/or accuracy in peanut leaf and root tissue samples from symptomatic and asymptomatic plants via DAS-ELISA, RT-PCR, and RT-qPCR. In addition, this study also used DAS-ELISA and RT-qPCR to quantitate TSWV loads in both tissue types from symptomatic and asymptomatic plants to assess if root tissue is a better TSWV reservoir than leaf tissue.

## 2. Results

### 2.1. Detection of TSWV in Leaf and Root Tissues of Symptomatic Plants

**Figure 1:**
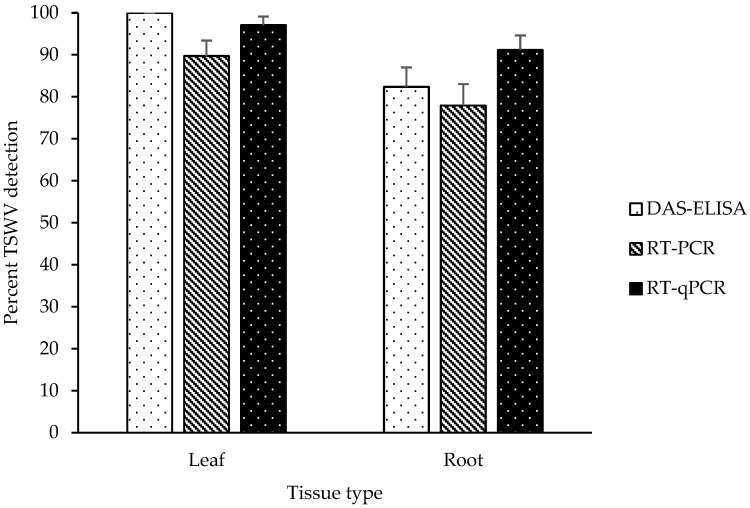
Mean percentage (±SE) of TSWV infection ascertained via DAS-ELISA, RT-PCR, and RT-qPCR in leaf and root tissue samples (n = 68 for each tissue type). Data were pooled across 2018 and 2019. TSWV symptomatic leaves at the growing points and the primary root of the same plants were sampled for TSWV detection.

TSWV detection in symptomatic leaf tissue samples was 100.00%, 89.71%, and 97.06% via DAS-ELISA, RT-PCR, and RT-qPCR, respectively (Figure 1). TSWV detection in root tissue samples from symptomatic plants by DAS-ELISA, RT-PCR, and RT-qPCR was 82.35%, 77.94%, and 91.18%, respectively (Figure 1). TSWV detection was significantly affected by detection method (F_2,401_ = 3.17, *p* = 0.0432) but did not vary by tissue type (F_1,401_ = 0, *p* = 0.9888), and the interaction (tissue type × detection method) effect was not significant (F_2,401_ = 0.03, *p* = 0.9701). The detection of TSWV in symptomatic plants was high for all three methods across tissue types. 

### 2.2. Detection of TSWV in Leaf and Root Tissues of Asymptomatic Plants

**Figure 2:**
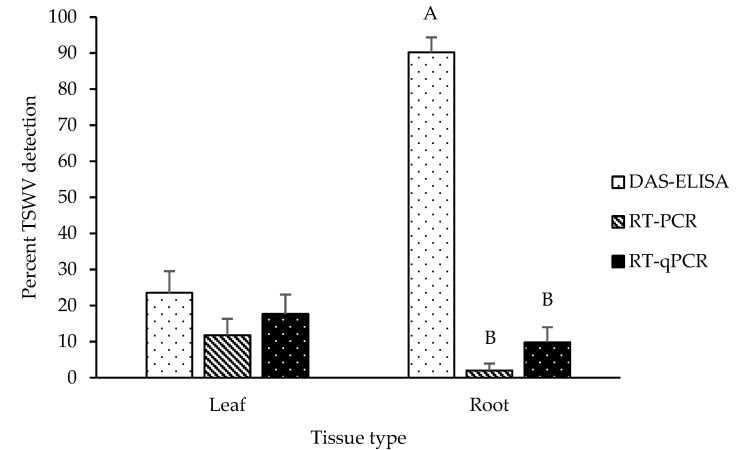
Mean percentages (±SE) of TSWV infection ascertained via DAS-ELISA, RT-PCR, and RT-qPCR in leaf and root tissue of asymptomatic peanut plants (n = 51 for each tissue type). Data were pooled across 2018 and 2019. Leaf at the growing points and the primary root of the same plants were sampled for TSWV detection.

TSWV detection in leaf tissue samples from asymptomatic plants was 23.53%, 11.76%, and 17.65% by DAS-ELISA, RT-PCR, and RT-qPCR, respectively (Figure 2). Detection of TSWV in root tissue samples from asymptomatic plants was 90.20%, 1.96%, and 9.80% for DAS-ELISA, RT-PCR, and RT-qPCR, respectively (Figure 2). A significant interaction effect between detection method and tissue type on TSWV detection was found (F_2,299_ = 16.63, *p* < 0.0001). TSWV detection percentages for the three detection methods were compared within each tissue type. TSWV detection in leaf tissue from asymptomatic plants was not different among the three detection methods (F_2,299_ = 1.25, *p* = 0.2877) (Figure 2). However, percent detection of TSWV significantly varied by detection method in root tissue from asymptomatic plants (F_2,299_ = 24.64, *p* < 0.0001). TSWV detection by DAS-ELISA was significantly higher than RT-PCR and RT-qPCR (Figure 2). The mean percentage of TSWV detection via DAS-ELISA was 46 times higher than RT-PCR and nine times higher than RT-qPCR.

### 2.3. TSWV Accumulation in Leaf and Root Tissues of Symptomatic and Asymptomatic Plants by DAS-ELISA and RT-qPCR

**Figure 3:**
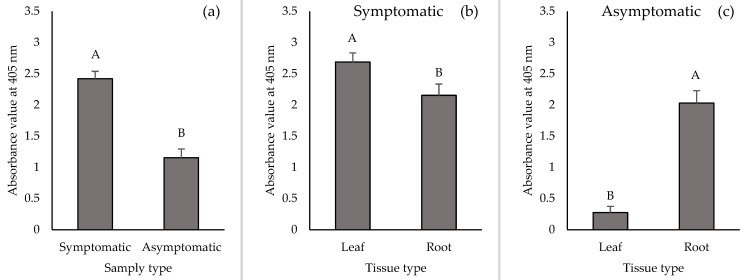

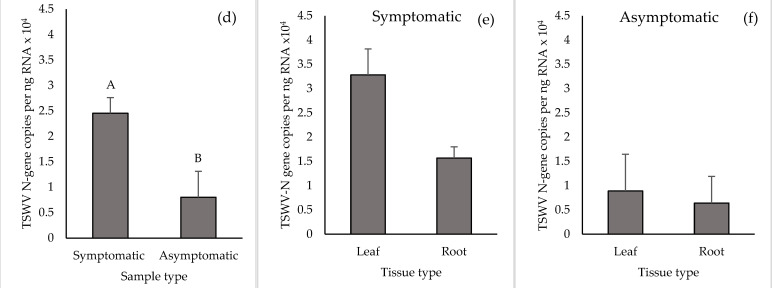
TSWV accumulation indicated by mean absorbance values at 405 nm from DAS-ELISA in (**a**) symptomatic (n = 136) and asymptomatic plants (n = 102) across tissue types and in leaf and root tissue samples from (**b**) symptomatic plants (n = 68 for each tissue type) and (**c**) asymptomatic plants (n = 51 for each tissue type). TSWV accumulation was also measured by RT-qPCR obtaining TSWV N-gene copies in (**d**) symptomatic (n = 128) and asymptomatic plants (n = 14) across tissue types and in leaf and root tissue samples from (**e**) symptomatic plants (n = 66, 62 for leaf and root samples, respectively) and (**f**) asymptomatic plants (n = 9, 5 for leaf and root samples, respectively). Data were pooled across 2018 and 2019.

TSWV loads in leaf and root tissue samples from symptomatic and asymptomatic plants were compared by DAS-ELISA and RT-qPCR. Overall, TSWV loads were higher in symptomatic than asymptomatic plant samples irrespective of tissue type based on absorbance values from DAS-ELISA (F_1,233_ = 58.78, *p* < 0.0001) (Figure 3a) and TSWV N-gene copies from RT-qPCR (F_1,137_ = 11.05, *p* = 0.0011) (Figure 3d). TSWV loads were significantly higher in leaf tissue samples than root tissue samples from symptomatic plants via DAS-ELISA (based on absorbance values) (F_1,133_ = 5.25, *p* = 0.0235) and numerically higher via RT-qPCR (Figure 3b, 3e). For asymptomatic plants, TSWV loads were more than seven times higher in root than leaf tissue samples based on absorbance values via DAS-ELISA (F_1,99_ = 62.48, *p* < 0.0001) (Figure 3c). However, TSWV N-gene copies in leaf and root tissue samples from asymptomatic plants were not different (Figure 3f).

## 3. Discussion

The evaluation of TSWV incidence in peanuts is commonly accomplished by visual assessments of typical TSWV-induced spotted wilt disease symptoms, such as yellowing, concentric ring spots, and stunting [1,9]. Foliar symptom-based screening can be confounded by biotic factors such as infection by other pathogens, arthropod infestation, TSWV resistance status, and timing of infection. For example, the infection of impatiens necrotic spot virus or peanut mottle virus can produce foliar symptoms akin to TSWV infection in peanuts [51,52,53]. Abiotic factors such as environmental conditions and chemical injury can also lead to foliar symptoms that resemble TSWV infection [54,55,56]. In such instances, it is useful to confirm TSWV infection with either a serology-based detection technique, such as DAS-ELISA, or a nucleic acid-based detection technique, such as RT-PCR. A few previous studies found that TSWV was more often detected by DAS-ELISA in peanut root samples than leaf tissue samples [16,21,25,27]. However, DAS-ELISA is prone to producing false positives when unstandardized tissue types are used, and it is unclear whether the high frequency of TSWV detection in root tissue samples reflected true TSWV incidence or was an overestimation due to false positives.

In this study, TSWV infection in leaf and root tissue samples from symptomatic and asymptomatic peanut plants was assessed by DAS-ELISA, RT-PCR, and RT-qPCR. TSWV accumulation in leaf and root tissue samples was also quantitated using RT-qPCR. The results from this study show that TSWV detection did not vary between leaf and root tissue samples or among the three detection methods in each tissue type from symptomatic plants. In addition, symptomatic leaf tissue samples had higher TSWV loads than root tissue samples from symptomatic plants. Dang et al. (2009) [22] also found congruency in TSWV detection between DAS-ELISA and RT-PCR while using peanut root tissue samples. However, it was not clear if the root tissue samples used for detection were collected from symptomatic and/or asymptomatic plants. Additionally, that study did not compare TSWV detection between root and leaf tissue samples.

In general, TSWV detection in asymptomatic leaf and/or root tissue samples was significantly lower than in symptomatic tissue samples. For asymptomatic plants, this study found similar percentages of detection between DAS-ELISA, RT-PCR, and RT-qPCR in leaf tissue samples, but the percentage of detection varied by method in root tissue samples. TSWV detection via DAS-ELISA was significantly higher than detection by RT-PCR and RT-qPCR in root tissue samples of asymptomatic plants. The inconsistency in TSWV detection between detection methods using root tissue samples from asymptomatic plants could, in part, be explained by two possible scenarios: either DAS-ELISA overestimated TSWV infection (false positives), or RT-PCR and RT-qPCR underestimated TSWV infection (false negatives). Low detection sensitivity of a detection assay could cause underestimation of TSWV infection. Sensitivity can be defined as the capability of the method/assay to reliably detect the lowest number of pathogen copies per test sample [57]. Generally, the sensitivity of nucleic acid-based detection assays with the use of gene-specific primers is higher than serology-based assays [39,57,58]. While the sensitivity of DAS-ELISA and RT-PCR has not been evaluated and compared specifically for TSWV detection in peanuts, such comparisons have been documented in other crops and pathosystems. The results from multiple studies indicate that the sensitivity of PCR can range from 2 to 625-fold higher than ELISA [59,60,61,62,63,64]. 

The reliability of ELISA can be affected by factors that lead to inaccurate detection results, even though ELISA is generally sensitive and specific with the use of monoclonal antibodies [37]. Well-recognized causes of inaccurate detection results include non-homogenous virus distribution in plants, interference of plant extracts, failure to detect certain virus serotypes, and cross-reactivity with other closely related viruses [50]. In addition, physiological and biochemical characteristics of the host plant and tissue type chosen for virus detection are important factors that are known to interfere with serological reactions in ELISA [47,48,49,65]. Non-specific antigen-antibody interaction due to the presence of plant proteins could lead to false positives in ELISA [62,66]. Detection of potato leafroll virus (PLRV) in potato tubers via DAS-ELISA resulted in a 70% overestimation of PLRV infection due to false positives [65]. Gunn and Pares (1988) [65] speculated that the false positives could have been caused by non-specific antibodies originating from co-purification of non-virus antigens (i.e., plant proteins) along with PLRV in the PLRV-specific antibody production process. While blocking reagents, such as ovalbumin, bovine serum albumin (BSA), and polyvinylpyrrolidone (PVP) are typically included in the commercial DAS-ELISA kits to reduce non-specific reactions, false positives could still arise from non-virus antigen-antibody interactions. Plant proteins such as pathogenesis-related proteins, lectins, and sesquiterpenoids are often present in virus-infected plants, and these plant-originated proteins could induce non-specific binding in ELISA [65,67,68]. For example, non-specific reactions in DAS-ELISA for cucumber mosaic virus detection in ornamental plants and wild weed species resulted in false positives [48,49]. Those studies demonstrated that components in plant extracts bound to microtiter plates and interacted with antibodies even under conditions that were unsuitable for antigen binding (i.e., the neutral pH and the presence of Tween) [48,49]. Plant roots are known to secrete chemical compounds, such as phenolics, terpenoids, and associated secondary metabolites, for defense against pathogenic microorganisms in soil [69]. In peanut root tissue, lectins are commonly present serving as defense proteins and are important for rhizobia agglutination in legume roots [70,71]. While such proteins could play a role in overestimating TSWV infection in peanuts, no empirical studies have been conducted to implicate their interference in DAS-ELISA-based detection.

Over- or underestimation of TSWV infection by DAS-ELISA could also be affected by the test threshold used. Threshold absorbance values to determine virus infection via DAS-ELISA have varied across studies ranging from two or three times the average absorbance value of negative controls (i.e., non-infected plant tissue) to the average absorbance value of negative controls plus three or four times the standard deviation [72]. It has been demonstrated that setting a more stringent detection threshold reduces false positives and increases false negatives. In one study where a range of threshold absorbance values was calculated using different methods, the lowest threshold value (0.040) resulted in 0.00% false negatives and 9.09% false positives, whereas the highest threshold values (0.131) resulted in 11.11% and 2.04% false negatives and false positives, respectively [72]. In this study, a high threshold value was selected using either a value of 0.1 or the mean of negative controls plus four standard deviations that were higher than 0.1. This stringent threshold value was used to help avoid false positives. In addition, both inarguably high and low absorbance values were obtained from root samples, with none of the values being ambiguous or close to the baseline threshold used in this study. If indeed DAS-ELISA was overestimating the TSWV infection in root tissue samples, it was likely due to non-specific antigen–antibody interactions rather than false positives related to threshold values.

Over- or underestimation of virus infection could also occur with nucleic acid-based detection assays. False-negative results commonly occur due to inhibitors interfering with the reaction by binding to DNA templates, interacting or competing with cofactor ions, and denaturing or degrading DNA polymerase [73]. Inhibitors are substances that are unintentionally extracted along with the nucleic acids of test samples; examples include phenolic compounds and heavy metals from the environment, cell debris, and residual reagents from the extraction procedure (e.g., phenol, EDTA, ethanol, and isopropanol) [47,74]. The housekeeping gene (alcohol dehydrogenase Class III) expression levels, assessed in terms of cycle threshold values, were lower with leaf tissue samples than root tissue samples suggesting that PCR inhibitors could be associated with root tissue samples (Table S1). Nevertheless, RT-PCR and RT-qPCR efficiently detected TSWV in root tissue samples from symptomatic plants using the same extraction method. Using column-based extraction can improve the purity of nucleic acid extracts; however, it can also increase the risk of losing nucleic acid extracts, which may lead to false negatives [47,75]. Loss of nucleic acids of the pathogen of interest can be especially problematic when the original quantity is relatively low, such as in asymptomatic plants. Asymptomatic plants generally accumulated less virus when compared with symptomatic plants [76,77]. This phenomenon was also observed in the current study, as TSWV loads were three times higher in symptomatic than asymptomatic tissue samples across tissue types. 

According to the RT-qPCR results, TSWV loads were higher in leaf tissue samples than in root tissue samples of symptomatic plants, while TSWV loads were not significantly different between leaf and root tissue samples of asymptomatic plants. The results indicate that it should be easier to detect TSWV in leaf tissue samples than root tissue samples of symptomatic plants due to higher TSWV accumulation in leaf tissue. When detecting TSWV in asymptomatic plants, leaf tissue samples are a better choice than root tissue samples, especially when DAS-ELISA is used, due to the possibility of false positives with root tissue samples.

This study demonstrated that DAS-ELISA might not be suitable for TSWV detection in peanut root tissue samples and could potentially lead to overestimation of TSWV infection. Nevertheless, DAS-ELISA is a reliable TSWV detection assay for peanut leaf tissue samples and good for large-scale screening in symptomatic/asymptomatic plants if an appropriate tissue type is used. In fact, it is possible to overestimate TSWV incidence in root tissue samples on some occasions if DAS-ELISA is used, possibly due to non-specific plant antigen-TSWV antibody interactions. Conversely, there is potential for underestimating TSWV in root tissue samples using RT-qPCR if PCR inhibitors are present.

## 4. Materials and Methods

### 4.1. Sample Collection

Samples of leaf and root tissue from TSWV symptomatic and asymptomatic peanut plants were used for TSWV detection. Samples were collected from four peanut fields established at the University of Georgia research farms in Tifton and Attapulgus, GA, in 2018 and 2019. From symptomatic plants, peanut leaves with distinct spotted wilt symptoms at growing points (top 10–15 cm) were collected. Leaf tissue samples from asymptomatic plants were randomly collected at growing points. Sections (~10 cm) of the primary root (i.e., taproot) cut immediately below the crown of the plants from which leaf samples were collected were used for TSWV detection. Samples of leaf and root tissue were placed in resealable bags and transported to the laboratory with ice packs. Field-collected samples were stored at 4 °C for up to five days before being processed and tested by DAS-ELISA. Afterwards, field samples were stored at −80 °C and later tested by RT-PCR and RT-qPCR.

In 2018 and 2019, samples of leaf and root tissue from TSWV symptomatic plants and asymptomatic plants of cultivars Georgia-06G Georgia Green, Georgia-16HO, and TUFRunner 511 [78,79,80,81] were collected. Leaf and root tissue samples from symptomatic plants were collected from 35 to 91 days post-planting (DAP). Conversely, leaf and root tissue samples from asymptomatic plants were collected at ~140 DAP. Samples were collected from peanut fields at the Ponder Farm in Tifton, GA and from the UGA Research Station at Attapulgus, GA. All other sampling details are elaborated in Table S2.

### 4.2. Detection and Quantitation of TSWV in Leaf and Root Tissue Samples by DAS-ELISA 

Samples of leaf and root tissue from symptomatic and asymptomatic plants were tested for the presence of TSWV using DAS-ELISA. TSWV loads in leaf and root tissue samples were quantitated by DAS-ELISA.

DAS-ELISA was performed in 96-well microtiter plates. A positive control (frozen, symptomatic leaf tissue of field-grown tobacco plants) and a negative control (leaf tissue of greenhouse-grown, non-infected peanut plants) with two replications each were included in each plate along with test samples. For both leaf and root tissue samples, approximately 0.03 g of tissue samples were ground in 300 µL of sample extraction buffer. Primary (anti-TSWV IgG) and secondary (anti-TSWV IgG conjugated with alkaline phosphatase) antibodies were used at a 1:200 dilution ratio (Agdia, Elkhart, IN, USA). The assay steps, including enzyme coating, incubation, and washing, were followed as per the manufacturer’s instructions. An hour after adding the substrate, final absorbance values were measured by a photometer at 405 nm (model Elx 800, Bio-Tek, Kocherwaldstr, Germany). Samples were considered positive when the final absorbance value was greater than the threshold value of the average absorbance value of negative control samples plus four standard deviations. To be more stringent, a value of 0.1 was adopted to define positive samples when the calculated threshold value was less than 0.1.

### 4.3. Detection and Quantitation of TSWV in Leaf and Root Tissue Samples by RT-PCR and RT-qPCR 

Samples of leaf and root tissue from symptomatic and asymptomatic plants were tested for the presence of TSWV using RT-PCR and RT-qPCR. TSWV loads in leaf and root tissue samples were quantitated by RT-qPCR.

For RT-PCR and RT-qPCR, total RNA from leaf and root tissue samples was extracted using an RNeasy plant mini kit (Qiagen, Valencia, CA, USA) according to the protocol provided by the manufacturer. Approximately 0.1 g of leaf or root tissue samples was used for RNA extraction. The concentration and the purity of the total RNA extracts were measured using NanoDrop^TM^ 2000 spectrophotometer (Thermo Fisher Scientific, Waltham, MA, USA). Complementary DNA was synthesized using the total RNA extracts (normalized to 50 ng/µL) with a Go-Script reverse transcription system (Promega Corporation, Madison, WI, USA) following the manufacturer’s instructions. Oligo dT primers were used for cDNA synthesis, and cDNA from each sample was used as a template for RT-PCR and RT-qPCR. A TSWV positive leaf tissue sample collected from an infected peanut plant in Tifton, GA, USA, was used as a positive control, and a leaf tissue sample collected from a non-infected, greenhouse-grown peanut plant was used as a negative control.

PCR was performed in a DNA engine thermocycler (Bio-Rad Laboratories, Hercules, CA, USA). The reaction mix included 5 µL of GoTaq Green Master Mix (Promega, Madison, WI, USA), 0.5 µL (0.5 mM) of each forward and reverse primer specific to the TSWV-N gene (forward: 5′-ATGTCTAAGGTTAAGCTC-3′; reverse: 5′-TTAAGCAAGTTCTGTGAG-3′) [22], 1 µL of synthesized cDNA, and the total reaction volume was adjusted to 10 µL by adding nuclease-free water. The PCR program started with an initial activation step at 95 °C for 15 min, followed by 35 cycles of 94 °C for 1 min, 52 °C for 45 s, and 72 °C for 1 min with a final extension step at 72 °C for 10 min. The presence of the targeted amplicons (~800 bp) was visualized by agarose gel (1%) electrophoresis.

The cDNA from leaf and root tissue samples was used for TSWV quantitation. Quantitative PCR was performed in a Realplex Mastercycler (Eppendorf, Enfield, CT, USA). The reaction mixture consisted of 2 µL of synthesized cDNA, 12.5 µL of GoTaq qPCR 2X MasterMix (Promega, USA), 0.5 µL of each forward and reverse primers (0.2 mM), and the final volume was brought to 25 µL by adding nuclease-free water. A pair of TSWV-N gene-specific primers were used (forward: 5′-GCTTCCCACCCTTTGATTC-3′; reverse: 5′-ATAGCCAAGACAACACTGATC-3′) [44]. Each sample had two technical replicates, including test samples and positive and negative controls. The reaction program started with an initial step of 95 °C for 2 min, followed by 40 cycles of 95 °C for 15 s, 55 °C for 60 s, and 72 °C for 20 s. The reaction program was extended with a melting curve analysis, which involved incubating the reaction mix at 95 °C for 15 s, 60 °C for 15 s followed by increasing the temperature by 0.5 °C per min for 20 min with a final step of 95 °C for 15 s. Melting curve analysis was used to rule out the non-specific binding of primers. TSWV loads in the samples were quantitated using the standard curve protocol with plasmids carrying TSWV-N gene inserts described by Shrestha et al. (2013) [30].

### 4.4. Statistical Analyses

Whether TSWV detection varied by tissue type and/or detection method was determined for samples from both symptomatic and asymptomatic plants. Data from symptomatic and asymptomatic plants were analyzed separately. The experiment was a two-way factorial design. Data were subjected to generalized linear mixed model analysis using PROC GLIMMIX procedure with a binomial distribution and the logit link function in SAS (SAS Enterprise 9.4, SAS Institute, Cary, NC, USA). Tissue type and detection method were fixed effects, while year and replication were random effects. The least-square means were used to identify differences in TSWV detection at α = 0.05 significance level with Tukey–Kramer adjustment.

TSWV loads were compared between symptomatic and asymptomatic plants using absorbance values from DAS-ELISA and TSWV N-gene copies from RT-qPCR. Data were pooled across years and tissue types for comparison between symptomatic and asymptomatic plants and subjected to generalized linear mixed model analysis using PROC GLIMMIX procedure in SAS. The tissue type served as a fixed effect, while year and replication served as random effects. A normal distribution with the identity link function was used for absorbance values, while a negative binomial distribution with the log link function was used for TSWV N-gene copies. The least-square means were used to identify differences in TSWV loads between treatments at α = 0.05 significance level with Tukey–Kramer adjustment.

## Data Availability

Not applicable.

## Supplementary Materials

The following are available online at https://www.mdpi.com/article/10.3390/pathogens10111476/s1, Table S1: Housekeeping gene (alcohol dehydrogenase class III) expression levels in root and leaf tissue samples from asymptomatic plants, Table S2: Summary of samples tested by DAS-ELISA, RT-PCR, and RT-qPCR for TSWV.
