# Supplementary material for: Discrepancies in Serology-Based and Nucleic Acid-Based Detection and Quantitation of Tomato Spotted Wilt Orthotospovirus in Leaf and Root Tissues from Symptomatic and Asymptomatic Peanut Plants
Source: Pathogens. 2021 Nov 12;10(11):1476. doi: 10.3390/pathogens10111476 (PMC8624541; doi:10.3390/pathogens10111476)
Supplement: Supplementary file 1 [file pathogens-10-01476-s001.zip › pathogens-1383516-supplementary.pdf]

Supplementary Materials

Discrepancies in Serology-Based and Nucleic Acid-Based Detection and Quantitation of Tomato Spotted Wilt Orthotospovirus in Leaf and Root Tissues from Symptomatic and Asymptomatic Peanut Plants

RT-qPCR efficiency evaluation using a housekeeping gene

The efficiency of RT-qPCR for leaf and root tissue samples were evaluated by using a house keeping gene of peanut. Primers for alcohol dehydrogenase class III were used (forward: 5’- GACGCTTGGCGAGATCAACA-3’; reverse: 5’- AACCGGACAACCACCACATG -3’). RT-qPCR was conducted using the protocol described in the main manuscript. Two subsets of six asymptomatic plant samples (n = 12) from 2019 were used for RT-qPCR. Ct values for leaf and root tissue samples were reported in Table S1. Three technical replicates were used for each sample. Overall, RT-qPCR efficiency was lower in root tissue samples than leaf tissue samples as the mean Ct value was higher in root than leaf tissue samples by 5 cycles.

Table S1. Housekeeping gene (alcohol dehydrogenase class III) expression levels in root and leaf tissue samples from asymptomatic plants

Subsets	Asymptomatic plants	Ct	
		Root	Leaf
I	1	27.5	24
	2	28.8	23.5
	3	27.8	25.4
	4	28.6	26.8
	5	28.8	23.7
	6	32.8	25.2
II	1	30.5	23.2
	2	31.9	25.3
	3	29.9	23.7
	4	28.3	25
	5	31.7	27.6
	6	34.1	24.3
	Mean (± SE)	30.1 ± 0.6	24.8 ± 0.4

Table S2. Summary of samples tested by DAS-ELISA, RT-PCR, and RT-qPCR for TSWV

Sample type	Year	N	tissue type	Number of positive			Percent positive		
				DAS-ELISA	RT-PCR	RT-qPCR	DAS-ELISA	RT-PCR	RT-qPCR
Symptomatic	2018	20	Leaf	20	19	20	100.00%	95.00%	100.00%
			Root	14	10	15	70.00%	50.00%	75.00%
	2019	48	Leaf	48	42	46	100.00%	87.50%	95.83%
			Root	42	43	47	87.50%	89.58%	97.92%
	Overall	68	Leaf	68	61	66	100.00%	89.71%	97.06%
			Root	56	53	62	82.35%	77.94%	91.18%
Asymptomatic	2018	9	Leaf	7	3	3	77.78%	33.33%	33.33%
			Root	8	0	1	88.89%	0.00%	11.11%
	2019	42	Leaf	5	3	6	11.90%	7.14%	14.29%
			Root	38	1	4	90.48%	2.38%	9.52%
	Overall	51	Leaf	12	6	9	23.53%	11.76%	17.65%
			Root	46	1	5	90.20%	1.96%	9.80%
